# Afterload quantitation for evaluation of myocardial strain

**DOI:** 10.1186/1532-429X-16-S1-P320

**Published:** 2014-01-16

**Authors:** Nathaniel Reichek, Danielle Janosevic, Meghana Jayam, Tazim Merchant, Madhavi Kadiyala, Simcha Pollack, Jie J Cao

**Affiliations:** 1 Cardiac Imaging Program, St. Francis Hospital-the Heart Center, Roslyn, New York, USA; 2 Research Department, St. Francis Hospital-the Heart Center, Roslyn, New York, USA; 3 Internal Medicine, Stony Brook University, Stony Brook, New York, USA; 4 Biomedical Engineering, Stony Brook University, Stony Brook, New York, USA

## Background

Systolic myocardial strain is load dependent, but the CMR literature largely disregards effects of myocardial afterload on strain and strain rate. This may reflect well known limitations of conventional myocardial afterload assessment using wall stress analyses, which are based on erroneous assumptions about left ventricular(LV) geometry and/or myocardial material properties. Therefore, we compared the utility of a nongeometric afterload index(NGI) derived from LV pressure(P) volume(V) and mass, which requires no assumptions about material properties, to that of conventional noninvasive end-systolic circumferential stress(CWS) as determinants of CMR LV circumferential strain(CST), ejection fraction(EF) and strain rate(SR) in normals(NL) and patients with nonischemic dilated cardiomyopathy(CM).

## Methods

We obtained breath-hold volumetric short-axis SSFP cines, cuff systolic P, systolic duration, LVV, M and EF, feature-tracking global CST(TomTec Imaging Systems) and mean SR in NLs(n = 39, 46%female, age 54.6(sd14.6)yrs) and CM (n = 35, 23% female, age 50.8(sd15.0) yrs, EF 27.2%(sd10.8%). CWS was calculated using Mirsky's formula(Biophys. J.1969) while NGI was determined as end-systolic PV/M.

## Results

EF, CST and CSR were markedly reduced in CM compared to NL(EF 27.2%(sd10.8)vs 58.4%(4.6), (-53%)p < 0.0001; CST -10.7%(5.3) vs -23.9%(4.3), (-55%), p < 0.0001);(CSR -32.1%/s(14.8) vs -65.7(14.9) p < 0.0001). But CWS was also markedly elevated in CM versus NL(CWS 307.6(9.2) vs 176.2(42.1)x 103 dyn/cm2,(+75%), p < 0.0001). Thus afterload excess due to adverse LV remodeling, may account for most EF, strain and strain rate reduction in CM. However, PV/M was more markedly increased than CWS, (162.6(sd48.9) vs 84.4(18.4)(+93%) p < 0.0001) and correlated more closely and significantly with EF, CST and CSR than CWS in the expected inverse relationship in both NL and CM subgroups(Table 1). In stepwise regressions only PV/M was a significant correlate of EF, strain and strain rate in both subgroups.

**Table 1:** Afterload Indices Versus EF, Strain and Strain Rate

	n	Spearman r	p
CWS vs EF NL	39	-0.29	ns

PV/M vs EF NL	39	-0.59	< 0.0001

CWS vs EF CM	35	-0.40	< 0.02

PV/M vs EF CM	35	-0.63	< 0.0001

CWS vs CST NL	39	-0.28	ns

PV/M vs CST NL	39	-0.46	< 0.003

CWS vs CST CM	35	-0.18	ns

PV/M vs CST CM	35	-0.49	< 0.003

CWS vs SR NL	39	-0.28	ns

PV/M vs SR NL	39	-0.40	0.012

CWS vs SR CM	35	-0.19	ns

PV/M vs SR CM	35	-0.51	< 0.002

## Conclusions

Afterload excess due to adverse LV remodeling is an important determinant of reduced myocardial and LV chamber function in CM, making a major contribution to reductions in EF, CST and SR, but PV/M, a simple, nongeometric afterload index, is superior to conventional wall stress calculation as a quantitative afterload index.

## Funding

St. Francis Research Foundation.

